# Alpharetroviral Vectors: From a Cancer-Causing Agent to a Useful Tool for Human Gene Therapy

**DOI:** 10.3390/v6124811

**Published:** 2014-12-05

**Authors:** Julia D. Suerth, Verena Labenski, Axel Schambach

**Affiliations:** 1 Institute of Experimental Hematology, Hannover Medical School, Carl-Neuberg-Strasse 1, 30625 Hannover, Germany; E-Mails: suerth.julia@mh-hannover.de (J.D.S.); labenski.verena@mh-hannover.de (V.L.); 2 Division of Hematology/Oncology, Boston Children’s Hospital, Harvard Medical School, 300 Longwood Avenue, Boston, MA 02115, USA

**Keywords:** retrovirology, alpharetroviral vector, gene therapy, clinical translation, regulatory requirements, vector safety

## Abstract

Gene therapy using integrating retroviral vectors has proven its effectiveness in several clinical trials for the treatment of inherited diseases and cancer. However, vector-mediated adverse events related to insertional mutagenesis were also observed, emphasizing the need for safer therapeutic vectors. Paradoxically, alpharetroviruses, originally discovered as cancer-causing agents, have a more random and potentially safer integration pattern compared to gammaretro- and lentiviruses. In this review, we provide a short overview of the history of alpharetroviruses and explain how they can be converted into state-of-the-art gene delivery tools with improved safety features. We discuss development of alpharetroviral vectors in compliance with regulatory requirements for clinical translation, and provide an outlook on possible future gene therapy applications. Taken together, this review is a broad overview of alpharetroviral vectors spanning the bridge from their parental virus discovery to their potential applicability in clinical settings.

## 1. Introduction

Human gene therapy incorporating genome engineering to enhance cell functions has the potential to cure numerous life-threatening diseases, including severe combined immunodeficiencies and cancer. The evolutionary optimized ability to stably integrate DNA into cellular genomes makes retroviruses very attractive for permanent therapeutic cell modifications. During the last decades, retroviruses have been developed into valuable tools for human gene therapy with the focus on vectors derived from gammaretroviruses and lentiviruses. However, retroviral vector integration into the target cell genome can cause insertional activation of cellular proto-oncogenes, potentially leading to serious adverse events such as leukemia. This adverse effect of retroviral vectors on cellular integrity is termed genotoxicity and can be influenced by several factors, such as vector design and integration target site selection. In contrast to conventional gammaretroviral and lentiviral vectors, alpharetroviral vectors have comparatively neutral integration target site preferences, hence alpharetroviral vectors might be less genotoxic and therefore of therapeutic value. In the present review, we highlight previous alpharetroviral vector discoveries as well as current developments and the potential of alpharetroviral applications for future human gene therapy strategies.

## 2. History of (Alpha-) Retroviruses

**Figure 1:**
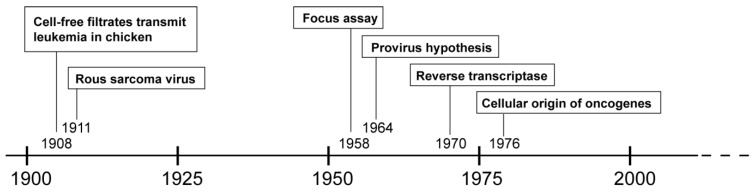
Timeline of hallmark events in the history of retrovirology.

More than 100 years ago, Vilhelm Ellermann and Oluf Bang demonstrated that cell-free filtrates were able to transmit leukemia in chickens [1]. A few years later, Francis Peyton Rous described the cell-free transmission of chicken sarcoma [2,3]. These groundbreaking discoveries indicated that cancer could be caused by viruses, a novel paradigm which was not widely accepted at that time. In fact, researchers supporting the concept of viruses causing cancer were said to either have “*holes in their heads or holes in their filters*” [4]. Nevertheless, especially the Rous sarcoma virus (RSV), a paradigmatic species of the alpharetroviral genus, played an important role in the history of retrovirology and in cancer research (Figure 1).

One important development made possible by the discovery of RSV was the focus assay, which provided the technical basis for studying viral infection on single viral particle and single cell levels and thus enabled researchers to investigate the molecular details of retroviral replication [5]. It was discovered that the RSV genome consisted of RNA [6], but that RSV infection required cellular DNA for replication [7]. This apparent contradiction was resolved with the provirus hypothesis published by Howard Temin in 1964: *“The results presented here with RSV can be most simply explained by the following model: virus enters a cell and directs formation of a DNA containing the genetic information of the virus. This new DNA, the provirus […], then acts as a template for formation of new nucleic acid, RNA, for the virion”* [8]. This hypothesis, independently proposed by Jan Svoboda and coworkers [9], was initially met with skepticism, but eventually became widely accepted upon identification of reverse transcriptase [10,11]. Many additional discoveries concerning the retroviral life cycle soon followed. However, the mechanism of RSV-induced cancer remained an unsettled issue until 1976, when molecular hybridization studies by Harold E. Varmus, J. Michael Bishop and coworkers revealed that, *“[…] part or all of the transforming gene(s) […] was derived from the chicken genome or a species closely related to chicken […]”* [12]. The transforming gene transferred by RSV was then identified as the *SRC* proto-oncogene, and soon followed by the discovery of additional oncogenes transferred by other retroviral species. Discovery of the cellular origin of viral oncogenes drastically changed the perspective of cancer research and immensely contributed to the understanding of cancer development. However, the transfer of proto-oncogenes was not the only mechanism through which retroviruses could cause cancer. In a process called insertional transformation, the retroviral promoter elements were shown to increase expression of cellular proto-oncogenes, such as *MYC*, thereby causing neoplasms, albeit as rare events and with much longer latencies than the acute transforming oncogene-transferring counterparts [13,14]. The genotoxic effect of retroviruses, first described in 1981, led to a severe setback in human gene therapy more than a decade later. Consequently, the mechanisms of insertional genotoxicity have been intensely studied and retroviral vector designs have been markedly improved, thus increasing the safety of human gene therapy. 

## 3. From the Virus to the Vector

### 3.1. Taking a Different Perspective: Retroviruses for Human Gene Therapy

The discovery of viral transfer of genes other than those required for viral replication [12] served as a paradigm for the development of human gene therapy vectors. With the increasing knowledge of the genetic origin of many diseases in the genomics era, researchers envisioned that by inserting a corrected version of a defective gene into a patient’s genome, a permanent cure could be achieved on a molecular basis. Gene therapy aims at curing life-threatening diseases, such as severe combined immunodeficiencies and cancer by exploiting the retroviral life cycle for the transfer of such therapeutic transgenes.

While aforementioned historical discoveries were made with avian-infectious alpharetroviral vectors, researchers working on gene therapy initially exploited gammaretroviral vectors derived from MLV (murine leukemia virus), which naturally replicates in mice. From a safety perspective, the availability of cell lines for the production of replication-defective retroviruses in 1983 marked an important step towards the development of retroviral vectors for human gene therapy [15]. Soon thereafter, pioneering studies in mice proved the concept of transferring genes into hematopoietic stem cells, albeit with low levels of gene transfer and gene expression [16,17]. During the next two decades, retroviral vectors were markedly improved with greater focus on vectors derived from gammaretroviruses (MLV) and lentiviruses (human immunodeficiency virus-1, HIV-1). Clinical benefit to previously terminally ill patients was demonstrated in several preclinical studies and clinical trials have employed these vectors to treat primary immunodeficiencies, such as severe combined immunodeficiency (SCID) and chronic granulomatous disease (CGD). However, despite therapeutic benefits, some of these patients suffered from severe adverse events due to clonal expansion of transduced cells, eventually leading to myelodysplastic syndromes and/or leukemias [18]. Furthermore, the clinical success in the CGD trial was hampered by the occurrence of vector silencing, which resulted in only a transient benefit [19,20]. These adverse events led to the temporary postponement of many gene therapy clinical trials. Intensive investigations revealed that they resulted from genotoxic integrations of retroviral vectors in the genome, leading to upregulation of cellular proto-oncogenes, such as *LMO2* and *MDS1-EVI1* [20,21,22,23,24]. First described for replicating retroviruses more than a decade earlier, the risk of insertional proto-oncogene activation had initially been anticipated to be low for replication-defective retroviral vectors. However, insertional activation still occurred in clinical trials, thus raising the urgent question of how to improve the safety of retroviral gene therapy. 

### 3.2. Genotoxicity of Retroviral Vectors

**Figure 2:**
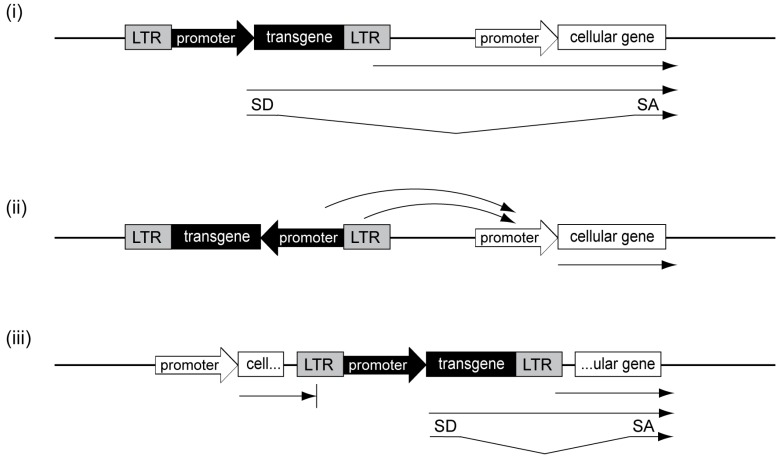
Genotoxicity mechanisms. (**i**) Promoter insertion upstream and in sense to cellular transcription units can lead to read-through transcription into adjacent cellular genes, either from the internal promoter or from the long terminal repeat (LTR) as indicated by the arrows. If splice acceptor (SA) and donor (SD) sites are present, promoter insertion can be accompanied by splice events; (**ii**) promoter activation is mediated by enhancer-interactions by respective elements in the internal promoter or in the LTR with cellular promoters; (**iii**) gene transcript truncation can lead to shortened cellular transcripts, either lacking 3' (left example) or 5' sequences (right example).

Identification of the mechanisms and reduction of underlying genotoxicity have become major objectives of the field of human gene therapy. In general, retroviral vector genotoxicity is due to upregulation of cellular proto-oncogene expression and can theoretically be caused by several mechanisms, such as (i) promoter insertion; (ii) promoter activation and (iii) gene transcript truncation (Figure 2).

(i) The genotoxic mechanism of promoter insertion describes the insertion of promoter sequences directly upstream of cellular transcription units, thereby adversely influencing their expression (Figure 2i). This can either be mediated by read-through transcription from the inserted promoter into the adjacent gene or by a combination of read-through transcription and splicing events involving vector and cellular splice sites. In 1981, promoter insertion became the first described mechanism of insertional transformation for replicating alpharetroviruses and caused neoplastic transformation in birds. In the reported cases, the viral promoter, which resides in the 5' and 3' long terminal repeats (LTRs) of the retrovirus, caused read-through transcription into the cellular *MYC* proto-oncogene and was detected by the presence of virus-*MYC* fusion transcripts [13,14]. In addition to the overexpression of adjacent genes, promoter insertion can also lead to oncogene capture by replication-competent retroviral vectors and thus to the development of acute transforming viruses. These viruses have an enormous genotoxic potential as oncogene expression occurs irrespective of their integration sites. However, while promoter insertion can be induced by any retroviral vector harboring promoter elements, oncogene capture is a very rare event and is restricted to replication-competent vectors. Additionally, in contrast to RSV, most acute transforming viruses become replication-defective, as they lose part of their viral coding sequences and thus require helper viruses for infectious viral particle formation.

(ii) In most clinical trials, which used replication-defective gammaretroviral vectors, promoter activation was identified as the primary cause of transformation (Figure 2ii). Here, the powerful enhancer in the gammaretroviral LTR most likely caused upregulated expression of cellular proto-oncogenes, such as *LMO2* or *MDS1-EVI1* [20,21,22,23,24]. While the mechanism of promoter insertion is restricted to upstream and in sense-oriented integrations of the retroviral vector adjacent to the affected cellular transcription unit, enhancer-mediated promoter activation can occur at different orientations and loci up to several hundred kilobases from the insertion site [25,26].

(iii) There is increasing evidence that gene transcript truncation is also a safety concern (Figure 2iii). In contrast to promoter insertion, gene transcript truncation is caused by intragenic retroviral vector integrations. These intragenic integrations frequently occur in intronic regions, involve aberrant splicing events and result in a loss of either 5' or 3' sequences of cellular genes. When transcription initiates from an inserted promoter, 5' sequences of the affected cellular gene are lost (Figure 2iii; right example). In contrast, when transcription is initiated from a cellular promoter followed by read-through into the retroviral vector, loss of 3' sequences can be caused by premature polyadenylation at polyadenylation sites introduced within the vector (Figure 2iii; left example). In several murine studies using lentiviral vectors, gene transcript truncations of 5' or 3' sequences led to leukemia development by either removal of regulatory regions of proto-oncogenes [27] or by downregulation of full-length transcripts of supposedly haploinsufficient tumor suppressor genes [28]. Functional consequences of gene transcript truncation were also demonstrated in a clinical trial involving one patient suffering from β-thalassemia [29]. After lentiviral β-globin gene transfer, this patient showed therapeutic benefit largely attributed to a dominant clone, in which intragenic vector integration in *HMGA2* had occurred. This insertion led to a 3' gene transcript truncation, rendering the shortened mRNA insensitive to microRNA-mediated downregulation, thus upregulating *HMGA2*, which led to clonal expansion. While this benign clonal expansion has not resulted in a serious adverse event to date, and is actually associated with clinical benefit, it clearly demonstrates the potency of gene transcript truncation to contribute to clonal imbalance in a clinical setting.

Promoter insertion, promoter activation and gene transcript truncation are the three most prevalent mechanisms of retroviral vector genotoxicity described to date. While these mechanisms have been described individually for the sake of clarity, insertional mutagenic events show a higher layer of complexity. For example, a single integration may affect more than one gene and induce transcriptional deregulation by more than one mechanism simultaneously [30]. Importantly, our current understanding of genotoxicity mechanisms allows several prevention strategies to be envisioned. Since insertional deregulation of cellular transcription is dependent on the presence of strong promoter/enhancer sequences and splice sites, optimized vector design should omit these elements. In addition, integration target sites influence genotoxicity, with potentially detrimental integrations occurring near or within genes, especially proto-oncogenes and tumor suppressor genes. In this regard, genome-wide studies of retroviral DNA integration have elucidated distinct integration target site preferences for different retroviral vectors. While gammaretroviral vectors preferentially integrate in the proximity of transcription start sites, CpG islands, and genes with implications in cancer, lentiviral vectors tend to integrate within transcription units of actively transcribed genes [27,31,32,33,34,35,36,37,38,39,40,41]. These integration target site preferences are influenced by retrovirus-specific interactions of the retroviral integrase with cellular tethering factors, such as lens-epithelium-derived growth factor/p75 (LEDGF) in the lentiviral [42,43,44,45,46] and bromodomain and extraterminal domain (BET) proteins in the gammaretroviral context [47,48,49]. These tethering factors direct retroviral integrations to specific regions in the genome and thus largely contribute to integration target site preferences. There have been attempts to modify retroviral vectors and/or their tethering factors with the aim to obtain potentially safer integration characteristics [50,51,52,53,54,55,56]. However, some of these modifications suffered from reduced gene transfer efficacy or incomplete redirection of integration site preference and clinical applicability remains to be shown. Importantly, in contrast to gammaretroviral and lentiviral vectors, alpharetroviral vectors have a relatively neutral integration spectrum [57,58,59,60,61]. It is currently unknown whether alpharetroviral integration follows a pattern yet to be identified or if it occurs independently from tethering factors. Nevertheless, the comparatively neutral integration pattern of alpharetroviral vectors renders them less genotoxic [59,60,61] and thus increases their therapeutic value for future human gene therapy.

### 3.3. Retroviral Vector Safety Criteria

In order to comply with latest safety standards, retroviral vectors need to fulfill the following criteria: (i) replication-incompetence; (ii) low genotoxicity and (iii) low immunogenicity.

(i) Replication-incompetence is of paramount importance, as uncontrolled virus spread represents an enormous safety hazard not only in terms of potential oncogene capture, but also in terms of high vector doses. Patients undergoing hematopoietic gene therapy may be immunocompromised and thus unable to fight virus spread, which can lead to unpredictably high vector copy numbers and transductions of cells other than the initial target cells. Replication competent retrovirus (RCR) formation led to the development of T-lymphocyte neoplasms in a rhesus model of stem cell transplantation using gammaretroviral vectors. The resulting lymphoma cells contained very high vector copy numbers [62], clearly demonstrating that uncontrolled vector spread is associated with unpredictably high vector dose, thus contributing to genotoxicity. Furthermore, replication selects for viruses with smaller genomes, eventually resulting in viruses lacking part or all of their genomic information. These viral integrants are not only unable to confer any therapeutic effect, but also increase the genotoxic risks, as described for open reading frame-defective retroviral vectors [63]. The generation of replication-defective retroviruses can be achieved by using the split-packaging system for viral particle production. In this system, the retroviral genome is split into multiple sequence components, either provided by different plasmids (transient production) or integrated into different genomic loci (stable production). Irrespective of the production method used, these sequence components either contain the genetic information of the retroviral vector or of viral proteins needed for particle production. Importantly, only the retroviral vector sequence harbors the packaging signal and sequences required for reverse transcription and integration. Thus, the genetic information of the *trans*-complementing sequences, *i.e.*, for structural proteins, replication enzymes and envelopes, will not be selectively incorporated into retroviral particles, nor will they be reverse transcribed and integrated into the target cell genome. As a consequence, transduced target cells cannot produce progeny viral particles due to the lack of expression of retroviral proteins [64,65,66]. The split-packaging design does not only avoid virus replication, but also creates space for large transgene cassettes due to the removal of viral coding sequences from the genomic message. To prevent *de novo* RCR formation, it is advisable to avoid sequence homologies between the *trans*-complementing and retroviral vector sequences. Splitting the genomic information needed for viral protein expression into more than two components further decreases the likelihood of RCR formation.

(ii) Especially the occurrence of serious adverse events in human gene therapy clinical trials has underscored the urgent need for retroviral vectors with low genotoxicity. Intensive research efforts have demonstrated that in addition to the integration pattern, strong promoter and enhancer elements profoundly affect genotoxicity. Thus, to further “disarm” retroviruses, viral promoter and enhancer elements, which reside in the unique three (U3) regions of viral LTRs, should be removed [67,68]. Since these transcriptional elements drive the expression of the viral genomic information, they are usually very potent and can modulate expression of cellular genes. This can lead to deleterious insertional upregulation of proto-oncogenes by promoter insertion or activation. Most gene therapeutic applications require physiologic transgene expression levels, which can be achieved by removing viral promoter and enhancer elements by introducing a so-called self-inactivation (SIN) deletion and inserting an internal promoter of choice. This not only reduces the vector’s genotoxicity [27,69,70], but also its potential phenotoxicity, which can be caused by the ectopic overexpression of transgenes potentially interfering with cell function and proliferation [71]. Due to the absence of full-length genomic RNA in transduced cells, SIN vectors have the additional advantage of further diminishing the likelihood of RCR formation. Another safety measure to decrease the risk of insertional deregulation of cellular gene expression is the removal of splice sites from the vector, as these sites can lead to aberrant splicing events and are frequently involved in gene transcript truncations [27,28,29]. Interestingly, all of the aforementioned vector developments and their effects on genotoxicity were recapitulated in an extensive *in vivo* study in a tumor prone mouse model. In this model, it was shown that oncogene activation by promoter insertion had the highest genotoxic potential, leading to early onset of vector-induced transformations and was associated with the use of primitive vector configurations, which still contained intact LTRs. Introducing the SIN deletion and strong internal promoters frequently led to enhancer-mediated promoter activation. In contrast, use of weaker internal promoters in the SIN context significantly reduced the genotoxic potential, with a shift in the most prevalent genotoxicity mechanism from promoter activation to gene transcript truncation. Further reduction in genotoxicity was observed for advanced retroviral SIN vectors with weak internal promoters and insulators. However, these vectors were still capable of inducing cellular transformation via gene transcript truncation, thus clearly arguing for the additional removal of splice sites [63].

(iii) Retroviral vector immunogenicity can be provoked by expression of viral or therapeutic proteins in transduced cells leading to humoral and cellular immune responses against transgene- and/or retroviral vector-derived epitopes [72,73]. Thus, immunogenicity can hamper the long-term presence of gene-modified cells, which is critical for most gene therapy approaches. Therefore, even though retroviral vector-mediated immunogenicity has not led to serious adverse events to date, it still deserves attention. Consequently, viral coding sequences should be completely eliminated from the vector, when designing the split-packaging system.

### 3.4. Alpharetroviral Vector Developments

Compliance with the aforementioned retroviral safety criteria is necessary in order to take advantage of the comparatively neutral alpharetroviral integration pattern for clinical applications. The most widely used alpharetroviral vector system is the RCAS (replication-competent avian leukosis virus LTR with a splice acceptor) system [74,75]. RCAS is based on RSV, in which the captured *SRC* oncogene has been exchanged by a unique restriction site, via which a transgene of interest can be inserted. The original RCAS vector is replication-competent in avian cells (its natural host) and expresses the transgene from a spliced message. Due to its replication-competence, high viral titers can be achieved in avian cells. In order to transduce mammalian cells, the alpharetroviral envelope glycoprotein was replaced by envelope glycoproteins derived from other retroviruses in a process called pseudotyping. While this allows transduction of mammalian cells, it generally precludes alpharetroviral replication in mammalian cells due to several blocks in the life cycle (as reviewed by [76]). Nevertheless, for clinical applications, the potential for viral replication/mobilization should be entirely eliminated, and thus pseudotyped RCAS vectors are unlikely to fulfill current clinical safety criteria. However, they are extremely useful for nonclinical applications and have been successfully used to transduce rhesus macaque hematopoietic stem and progenitor cells. After transplantation, long-term polyclonal engraftment with gene marking in myeloid and lymphoid lineages was observed, thus clearly underlining the clinical relevance of alpharetroviral vectors [77]. RCAS-derived vectors lacking the envelope gene have been designed to facilitate the generation of variably pseudotyped alpharetroviral vectors. Pseudotyped alpharetroviral vectors can be produced to allow the transduction of mammalian cells by providing the genetic information for the envelope *in trans* in packaging cells [78,79]. Since the vectors lacking the envelope gene do not contain all the information needed for viral replication, these vectors can be considered replication-defective even in avian cells. However, they lack the advanced split-packaging design and the SIN deletion, which are present in state-of-the-art gammaretroviral and lentiviral vectors and which are required for clinical applications [80,81,82].

**Figure 3:**
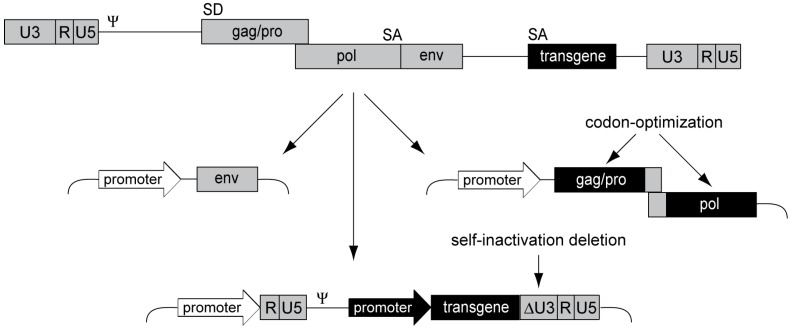
Alpharetroviral SIN split-packaging system. Schematic depiction of the alpharetroviral split-packaging system, with a replication-competent alpharetrovirus shown on top and the three respective split-packaging system components shown below. The viral coding sequence components are split onto two plasmids, one encoding the viral envelope (env) and one encoding the gag-pro/pol polyprotein. The latter was codon-optimized, sparing approximately 300 bp in the gag-pro/pol transition region to ensure proper frameshifting. The vector constitutes the third component of the split-packaging system. An external promoter drives the expression of the RNA, which contains an internal transgene expression cassette, a packaging signal (Ψ), and R, U5 and U3 regions, allowing for packaging, reverse transcription and integration. An alpharetroviral SIN vector was designed by removing transcriptional control elements from the U3 region (ΔU3) of the LTR.

Aiming for a clinically applicable alpharetroviral vector system, we designed an advanced split-packaging design completely avoiding sequence overlaps between the constructs expressing retroviral proteins *in trans* and the actual genomic message to be packaged [83] (Figure 3), thus minimizing the risk of RCR formation. The resulting transfer vector is also devoid of splice sites as the alpharetroviral splice donor is within the gag reading frame, further preventing potentially genotoxic splicing events. This is in contrast to gammaretroviral and lentiviral vectors, which still contain retroviral splice sites. Using the alpharetroviral split-packaging system, we successfully generated alpharetroviral particles from human 293T cells via transient plasmid transfection, albeit with low titers. However, codon-optimization of the alpharetroviral gag-pro/pol sequence for human tRNA codon-usage preferences spared ~300 bp in the gag-pro/pol transition region required for ribosomal frameshifting (Figure 3), and drastically increased titers by several orders of magnitude (approx. 10^7^ transducing units (TU)/mL) [83].

In a next step, we removed transcriptional control elements from the LTRs to generate alpharetroviral SIN vectors with reduced genotoxic potential and the ability to mediate physiologic transgene expression levels via internal promoters (Figure 3). Initially, we deleted 159 nucleotides of the wild-type U3 region, preserving the terminal 3' and 5' U3 sequences to maintain integrase attachment and polyadenylation sites [83]. While we showed that this initial SIN design reduced U3 transcriptional activity to background levels, this vector still contained the TATA box, a core promoter element of retroviruses. To exclude potential transcription due to this promoter element, we generated a second generation SIN vector by deleting the TATA box [60]. Importantly, both SIN designs eliminated several enhancer elements from the U3 region, such as CAAT enhancers, Y box motifs, and CArG boxes [84], thus increasing retroviral vector genosafety [69,85,86] and avoiding interference with the regulation of the internal promoter.

Using this latest generation of alpharetroviral SIN vectors, we and others demonstrated a comparatively neutral integration pattern in murine hematopoietic stem and progenitor cells, reduced genotoxicity in sensitive *in vitro* immortalization assays [60], and lack of aberrant splicing [87] compared to clinically used gammaretroviral and/or lentiviral SIN vectors. In more sophisticated integration site analyses in human hematopoietic stem and progenitor cells, alpharetroviral vectors exhibited a comparatively neutral integration pattern with regard to several annotated genomic features and potentially dangerous genomic loci, as well as with regard to epigenetically-defined functional genomic regions, such asenhancers (H3K4me1+) and promoters (H3K4me3+) when compared to gammaretroviral or transcribed gene bodies (H3K36me3) when compared to lentiviral vectors [61]. Finally, proof-of-principle was provided for the alpharetroviral genetic modification of hematopoietic stem and progenitor cells in mice [60], the modification of human T lymphocytes and NK cells (unpublished data), and phenotypic correction of X-linked CGD in a humanized mouse model [87].

In general, retroviral gene expression can be hampered by silencing, one of the cellular defense mechanisms against foreign DNA [88], leading to the downregulation of transgene expression. Silencing is mediated by cellular factors, which can recognize repressive elements on the vector, such as the retroviral primer binding site [89] or parts of the LTRs [90]. Silencing results in the formation of repressive epigenetic marks, including DNA methylation and histone deacetylation. In addition to gammaretro- and lentiviral vectors (reviewed in [91]), silencing has also been described in the context of alpharetroviral vectors [92,93,94,95,96,97,98]. It can generally be influenced by the target cell species [96], the differentiation status of the cell [99], the cell type [100] and the position of the integration site [101,102,103]. As alpharetroviral silencing has been reported to be more pronounced in mammalian than in avian cells [96], future modifications of alpharetroviral vectors should contain “antisilencing” modifications to ensure sustained therapeutic transgene expression. Such modifications include the removal of repressive elements from the vector and/or the insertion of supporting transcriptional regulatory elements. To this effect, the removal of viral coding sequences (split-packaging design) and the removal of transcriptional elements (SIN design) might already have eliminated repressive elements from the vector. In addition, transgene expression in SIN vectors is independent from LTR-mediated transcription and has been reported to be less susceptible to silencing in the context of gammaretro- and lentiviral SIN vectors (reviewed in [91]). Nevertheless, future incorporation of supporting transcriptional regulatory elements, such as scaffold/matrix attachment regions, insulators, locus control regions, ubiquitous chromatin opening elements [104] or chimeric versions thereof [105], is likely to enhance the therapeutic potential of alpharetroviral SIN vectors [76,106,107].

## 4. On the Road to Clinical Applicability: Production Perspectives of Alpharetroviral Vectors

In the last decade, the number of gene therapy clinical trials using gammaretro- and lentiviral vectors for stable genome modifications has almost doubled from 254 in 2004 to 498 in 2014. (http://www.abedia.com/wiley/; updated June 2014). Thus, there is an increasing demand for clinically applicable retroviral vector batches partially shifting the focus of successful clinical translation to economic vector production. 

In general, retroviral vectors can be produced either transiently or stably. Both methodologies possess distinct advantages and disadvantages regarding establishment, flexibility, reproducibility, up-scaling possibilities and quality assurance. For transient production, all components of the split-packaging system (the vector and the helper constructs encoding necessary viral proteins) are transiently delivered as plasmids into the packaging cells. This allows for flexible exchange of vector components. In contrast, the establishment of a stable production for clinical vectors is highly time-consuming and labor-intensive. Here, all components need to be stably integrated into the genome of the virus-producing packaging cell line followed by the identification of high-producer clones. Such clones usually display a high level of batch-to-batch product consistency, whereas transiently produced viral particles are subject to significant fluctuations [108,109]. Due to the obligatory delivery of good manufacturing practice (GMP)-grade plasmids, up-scaling of the transient production process is expensive and technically demanding. Additionally, transiently produced vector batches for clinical application need to be purified from remaining plasmids to avoid uncontrolled plasmid delivery to the patient. Taken together, the viral vector generation via stable packaging cell lines is preferred from the regulatory, the production-related and therefore from the economical point of view.

Irrespective of the production method, the formation of RCR is a major safety concern. RCR formation can result from recombination events between the vector and either the helper constructs or endogenous retroviruses [110] within the producer cell. Therefore, intensive screening for RCR is required for clinical vector batches, the producer cells as well as for treated patients [111]. In general, the likelihood of RCR-formation is lower in stable packaging cell lines compared to transient vector production, because stable producer cells are not transfected with millions of plasmid molecules [112]. Regarding the probability of RCR formation, alpharetroviral vectors possess additional safety features compared to other retroviral vector platforms with a split-packaging design, as alpharetroviral vectors do not exhibit any sequence overlap with their helper constructs encoding alpha gag-pro/pol and the envelope glycoprotein (see Section 3.4). This is of importance, as an overlap of as little as 8 bp already increases the probability of recombination events and, ultimately, the formation of RCR [113]. Moreover, alpharetroviruses can efficiently infect but generally not replicate in mammalian cells, probably due to various blocks in the retroviral life cycle [75,114] (as reviewed by [76]). Therefore, even in the improbable case of RCR-formation, the newly formed virus is not likely to propagate in subjects of human gene therapy trials. Of note, RCR-formation was never detected despite intensive PCR-based screening in the follow-up analysis of patients receiving retrovirally-modified cells [115].

Whereas a number of different stable packaging cell lines of murine and human origin are available for gammaretroviral vectors, the establishment of such cell lines in the lentiviral context is still challenging. Long-term expression of certain lentiviral packaging components (e.g., the protease) is cytotoxic, leading to silencing and loss of productivity over time [112,116]. Therefore, transient production of clinically applicable lentiviral vectors is currently the technology of choice. 

The first stable packing cell line (Isolde) for alpharetroviruses with an advanced split-packaging design was developed in 1990 by Cosset and colleagues [117]. Here, the RSV-derived viral genome was split onto three distinct plasmids with individual antibiotic resistance expression cassettes. To avoid packaging and replication of the helper constructs (encoding for alpha gag/pol and the natural envelope (subgroup A)), the packaging signal ψ as well as the 3'LTR- regions were deleted. All constructs were serially transfected into QT6 cells (a quail fibrosarcoma cell line) and selected via antibiotic treatment, yielding titers of up to 10^5^ TU/mL. Although all constructs possessed shared homologous regions, formation of RCRs was not detected [117]. Because of a higher productivity [118], RCAS vectors and derivatives have usually been produced in DF-1 cells [119], an untransformed chicken embryo fibroblast-derived cell line, which does not contain endogenous viruses closely related to alpharetroviruses. After transient delivery of the replication-competent RCAS vector, spreading of the vector throughout the DF-1 culture occurs resulting in high titer retroviral batches. For RCAS vectors harboring transgenes ≤2.8 kb, titers of >10^7^ infectious units/mL were produced [114]. As mentioned above, the potentially replicative nature of the RCAS vector and the presence of gag/pol and env exclude this technology for human gene therapy approaches. Therefore, we established a replication-defective alpharetroviral SIN vector platform, derived from the RCASBP-Y DV vector [120], for clinical applications.

Using this vector platform, we transiently produced alpharetroviral SIN vector batches with titers in the range of 10^7^ TU/mL in human cells (293T) after optimization of the alpharetroviral gag-pro/pol nucleotide sequence for human tRNA codon usage [83] (see Section 3.4). The internal reporter gene expression cassette used in this experiment was relatively small with a size of ~1.8 kb. However, using the optimized gag-pro/pol we also successfully generated alpharetroviral particles with internal cassettes of ~5.8 kb at reasonable titers (10^6^ TU/mL). While the theoretical packaging capacity of alpharetroviral vectors is approx. 8.8 kb [121], which would allow incorporation of large therapeutic transgene cassettes, the actual production feasibility needs to be determined empirically, as the nature of the transgene can have a greater impact than the sheer sequence length [122].

With the codon-optimized alpharetroviral gag-pro/pol construct and the same internal reporter gene expression cassette used for transient transfection, we established stable, monoclonal alpharetroviral SIN packaging cell lines derived from human 293 cells (DSMZ, ACC 305) showing sustained productivity over at least six months (unpublished data). The titers varied depending on the glycoprotein used for pseudotyping and ranged from 10^6^ TU/mL (pseudotyped with RD114/TR [123]) to 10^7^ TU/mL (pseudotyped with ecotropic env). For future stable and economic alpharetroviral vector production, the capacity to culture 293 cells in suspension could greatly simplify the production process and thereby reduce the production costs [124]. Furthermore, suspension-growing producer cells lack extracellular matrix proteins, predominantly proteoglycans, which have been shown to inhibit retroviral transduction [125]. In addition to high titers of alpharetroviral vectors, another advantage of using human cells for viral particle production is the potentially reduced immunogenicity of the retroviral particles. Since the viral membrane envelope is derived from the producer cell, all incorporated membrane proteins have been processed in the packaging cell. Even though cellular proteins are only efficiently incorporated into viral particles when expressed at high densities at the plasma membrane [126], the possibility of cross-species transfer of membrane proteins should be excluded. Furthermore, viral proteins produced in human cells possess human glycosylation profiles and are therefore less immunogenic. This is especially important for *in vivo* gene therapy approaches [127], in which the viral particles are directly applied to the patient and, thus, are directly exposed to the patient`s immune system. However, this could also apply to *ex vivo* approaches, where the gene-modified cells, still harboring viral membrane-derived proteins on their outer surface, are delivered to the patient. The viral membrane-derived proteins on the modified cells could potentially be recognized and eliminated by the recipient’s immune system. In summary, alpharetroviral particles produced in human suspension cells might harbor improved immunological safety characteristics as well as a higher potency in terms of transduction efficacy.

Increasing demand for clinically applicable retroviral vector batches demonstrates the importance of economic vector production for future gene therapy trials. In contrast to transient vector generation, the use of stable packaging cell lines promises consistent, economic, large-scale vector production and thus potentially allows treatment of greater numbers of patients. The perspective of stable alpharetroviral SIN vector production together with several additional safety features, such as the lack of sequence overlap between the vector and the helper constructs as well as the general inability to replicate in mammalian cells, underline the potential value of alpharetroviral SIN vectors for future gene therapy approaches.

## 5. From Bench to Bedside: Regulatory Requirements for Clinical Translation

Gene therapy using retroviral vectors has proven to be very effective in several clinical trials targeting hematopoietic stem cells and T-lymphocytes. Previous trials primarily employed gammaretroviral and lentiviral vectors, but the knowledge gained from experiences with these retroviral family members can be applied to advance new generation alpharetroviral SIN vectors to the clinical arena.

Generally, regulatory agencies, e.g. the U.S. Food and Drug Administration (www.fda.gov) and the European Medicines Agency (www.ema.europa.eu), are responsible for reviewing and approving planned gene therapy clinical trials. In light of previous severe adverse events, it is imperative to prove that the intended alpharetroviral gene therapy strategy is safe. To meet this goal, several recommendations for advanced therapy medicinal products (ATMP, a therapeutic product based on genes, cells or tissues) have been established (see recommendations on aforementioned webpages). For the regulatory review process of ATMPs, careful risk-benefit assessments need to be performed in the context of the particular clinical indication under study. This includes characterization of the alpharetroviral vector, such as content (including the transgene), delivery mode, and the somatic cell therapy product’s behavior in different contexts (e.g., isolated tissues and living organisms). Kaufmann *et al.* [87] provided a good example of validation of alpharetroviral gene therapy for a specific disease in a preclinical mouse model employing transduced mouse hematopoietic stem cells and, as a second step, in a humanized mouse model using alpharetrovirally corrected patient cells. 

In general, design of alpharetroviral vectors for clinical applications should comply with replication-incompetence, low genotoxicity, low immunogenicity, and the exclusion of germ-line integrations. Also the following points should be taken into account for the transgene and its product (see FDA guidelines): (i) preferably localized or lineage-specific expression rather than systemic/ubiquitous expression; (ii) low level and duration of expression (to avoid phenotoxicity); and (iii) anticipation of acute *vs.* long-term effects. With respect to transgenes, especially growth factors, growth factor receptors [128] and immune modulators should be handled with care.

There are basically two different ways to deliver therapeutic transgenes into patient cells—*in vivo* or *ex vivo*. While *in vivo* transgene delivery is relatively simple since it only requires the injection of purified virus into the target organ or into the bloodstream, it is associated with several concerns including toxicity, immunogenicity, lack of specificity and dose-control. In contrast, *ex vivo* transgene delivery is more complex. It requires isolation of the target cells, *in vitro* culture, and re-infusion after cell modification. However, *ex vivo* transgene delivery enables a tight control of specificity, vector dose, and circumvents human complement inactivation of retroviral particles. As for alpharetroviral gene therapy, the *ex vivo* method is thus preferred. 

Preclinical investigation cannot be applied directly to patients because of regulatory requirements (except for the “hospital exemption clause/compassionate use”) and ethical concerns. Therefore, appropriate animal models have to be chosen, which should ideally be biologically relevant and should model the disease phenotype. In the case of a specific monogenetic disease, the corresponding knock out models should be used. Ideally, also the transplantation of patients’ cells in so called “humanized” mouse models, which accept human grafts, e.g., the NOD/*Prkdc^scid^*/*Il2rg* (NSG) knock out model, should be performed.

Candidate target cells for clinical application include human CD34+ hematopoietic precursor cells and T cells. Both cell types can be efficiently transduced with alpharetroviral vectors, as demonstrated by our group [60] (and unpublished data). Moreover, Kaufmann *et al.* [87] showed efficient transduction of X-linked chronic granulomatous disease (CGD) patient derived CD34+ cells with an alpharetroviral vector expressing a functional version of the defective gene. Importantly, upon transplantation of the gene-modified cells into the NSG mouse model, they demonstrated functional disease correction and long-term expression of the transgene without obvious side effects. Interestingly, no aberrant splicing, caused by interference of viral and cellular splice sites, was observed for alpharetroviral in contrast to lentiviral vectors, implying that the safety profile of alpharetroviral vectors may even extend beyond their more neutral integration pattern in clinically relevant human CD34+ cells [61,87]. 

In addition, some overall safety considerations of alpharetrovirally genetically modified cells as somatic cell therapy medicinal products need to be addressed *in vivo*. This includes the analysis of (i) biodistribution and toxicology (to identify potential sites of toxicity), (ii) immuno-toxicity/immunogenicity (vector/transgene products affecting the immune system, pre-exisiting immunity and cross-reactivity) and (iii) shedding issues of retroviral vectors [129]. Ideally, most analyses should be accomplished with the clinical vector production lot and, if possible, in a controlled environment and in GMP-like compliance. 

## 6. Potential Future Clinical Applications of Alpharetroviral SIN Vectors

The use of gammaretro- and lentiviral vectors enabled several gene addition strategies in clinical trials targeting human hematopoietic stem cells and T-lymphocytes. In these trials, retroviral transduction was performed *ex vivo* followed by transplantion of genetically engineered cells into patients suffering from inherited diseases, e.g., from severe combined immunodeficiency [130,131,132], Wiskott-Aldrich syndrome [24,133], chronic granulomatous disease [19,20], adrenoleukodystrophy [134] and metachromatic leukodystrophy [135], or from cancer (examples below). While some of the gene therapies were quite effective, side effects related to insertional transformation were also observed (see Section 3.2). These adverse events were primarily related to the first generation LTR-driven gammaretroviral vector architecture and the use of hematopoietic stem cells, which are more prone for insertional transformation than other cell types. Since the integration pattern can also influence the likelihood of insertional transformation, more randomly integrating vectors represent potentially safer tools for future gene therapy trials. Generally, we foresee three main arguments for the use of alpharetroviral vectors in clinical translation. (i) The aforementioned potentially safer and more random integration pattern that could provide a lower risk of insertional mutagenesis; (ii) a clean vector architecture, not only lacking viral enhancer, promoter and splice elements, but also viral coding sequences, which can potentially contribute to immune responses as observed in patients treated with *ex vivo*-engineered T-cells, leading to limited peripheral persistence [72,73]; and (iii) the stable packaging cell line production perspective for alpharetroviral vectors associated with an economic and consistent production of larger virus batches, as needed for treatment of higher number of patients or to deliver greater numbers of modified cells per patient. 

Relatively high cell numbers are needed for a number of T cell applications. Given the efficient transduction of primary T cells and the stable production perspective, alpharetroviral vectors are ideal tools for genetic modification of primary T cells, which is why we elaborate on the potentially promising applications here. T cells can be easily purified from the peripheral blood and have thus been a promising target for genetic modification for almost 25 years. The first trial of genetically modified T cells was performed by Rosenberg and coworkers [136], who demonstrated gammaretroviral transduction of tumor-infiltrating lymphocytes. During the past two decades, numerous promising trials with genetically modified T cells were initiated, taking advantage of antigen-specific (i) T cell receptors (TCRs) and (ii) chimeric antigen receptors (CARs) as well as (iii) suicide gene approaches. 

(i) In the TCR approach, T cells recognize tumor peptides by specifically isolated tumor-specific TCRs. Gammaretroviral vectors were introduced into T cells to encode the α and β TCR chains forming a TCR specific for the melanoma antigen MART1 [137], CEA (for colorectal carcinoma), NY-ESO-1 (melanoma), WT1 (leukemia), and CMV pp65 (for EBV treatment) [138,139,140]. 

(ii) Furthermore, T cells can be engineered to express a chimeric antigen receptor (CAR) comprising an extracellular single chain-antibody (scFv) linked to an intracellular CD3ζ domain (and optionally further co-stimulatory domains). In comparison to conventional TCRs, CARs are not HLA-restricted or dependent on antigen presentation. As for TCRs, a larger list of potential tumor targets has been developed [141] and corresponding scFv are available. Also, next generation CARs were developed exploiting new transmembrane domains and other co-stimulatory domains (e.g., CD28, 4-1BB) [142]. CAR-T cells have shown remarkable results in B cell malignancies [143,144,145] and neuroblastoma [146].

(iii) Another interesting avenue is T cell engineering with suicide genes, as in the clinical setting to control graft *vs*. host disease (GvHD). Several trials have documented the feasibility of the HSV TK (thymidine kinase) to serve as a suicide gene, which converts the clinically approved prodrug Ganciclovir into a toxic substance [147,148]. Recently, a chemically inducible safety switch, the inducible caspase 9 (iCasp9), was developed and proved to be very effective, eliminating >90% of modified T cells within 30 min after administration and resolving the GvHD without recurrence [149]. 

Interestingly and arguing in favor of further clinical trials, T cell applications seem to have a lower propensity for insertional transformation. Genetically engineered T cells (transduced by a gammaretroviral LTR-driven vector) were followed in patients for over 10 years without any overt genotoxicity or evidence of transformation [150]. This favorable risk assessment is also in agreement with recent evidence from mouse models showing a relative resistance of mature T cells to insertional transformation by retroviral vectors and even known T cell oncogenes [151,152]. Nevertheless, specific conditions have been described that caused insertional transformation in T cells [153]. In this regard, the more neutral integration pattern of alpharetroviral SIN vectors is expected to further decrease the likelihood of adverse events related to insertional mutagenesis. 

In addition to gene-addition strategies as discussed above, gene correction or gene disruption strategies, allowing for site-specific genome editing, can also be of great therapeutic value. Here, integrating and non-integrating alpharetroviral vectors could be useful for delivery of the required components to the target cell. Emerging technologies for site-specific genomic editing include Zinc finger nucleases, transcription activator-like effector nucleases, and the CRISPR/Cas9 system. These technologies exploit designer nucleases, which are relatively large or have to be used as pairs. Therefore, the theoretically large coding capacity of alpharetroviral vectors could be beneficial. 

In summary, several useful applications for gene therapy have been recently developed using gammaretro- and lentiviral vectors for genetic modification of hematopoietic cells. Given the more random and potentially safer integration pattern, the clean vector architecture and the stable packaging perspective, several potential alpharetroviral vector applications could be envisioned for future clinical use.

## 7. Conclusions and Outlook

Alpharetroviruses were discovered more than 100 years ago. However they have come into play as potential retroviral vectors for human gene therapy only very recently, when serious adverse events in clinical trials underscored the need for safer vectors. Intensive research efforts revealed that retroviral vector genotoxicity is largely influenced by the vector architecture and the integration pattern. Thus, alpharetroviral vectors, which have a relatively neutral integration pattern compared to clinically used gammaretroviral and lentiviral vectors, represent attractive and potentially safer tools for human gene therapy. We applied the latest concepts of retroviral vector design to develop state-of-the-art alpharetroviral SIN vectors with a split-packaging design. Proof-of-principle for these vectors was provided by the genetic modification of clinically relevant target cells, such as hematopoietic stem and progenitor cells and T-lymphocytes. Moreover, future incorporation of supporting transcriptional regulatory elements protecting from cellular silencing mechanisms is likely to further enhance the therapeutic potential of alpharetroviral vectors. Since upscaling retroviral vector production represents one of the major challenges on the road to clinical translation, it is especially promising that alpharetroviral SIN vectors can be produced transiently as well as from stable human packaging cell lines. Compliance with several regulatory requirements, including performance of preclinical biodistribution and toxicology studies of the gene therapy product and careful risk-benefit assessments are still needed to further advance alpharetroviral vectors to the clinical arena. Altogether, the reduced genotoxicity in combination with the perspective of economic, stable vector production underlines the potential of alpharetroviral SIN vectors as useful tools for future human gene therapy strategies. Thus, as paradoxical as it seems, a viral genus, first noted for its transforming potential, might contribute to safer human gene therapy in the near future.

## References

[1] Ellermann V., Bang O. (1908). Experimentelle Leukämie bei Hühnern. Zentralbl. Bakteriol. Parasitenkd. Infektionskr. Hyg. Abt. I..

[2] Rous P. (1910). A transmissible avian neoplasm. (sarcoma of the common fowl.). J. Exp. Med..

[3] Rous P. (1911). A sarcoma of the fowl transmissible by an agent separable from the tumor cells. J. Exp. Med..

[4] Diamond L., Wolman S.R. (1989). Charlotte friend, ph.D. 1921–1987. A scientist’s life. Ann. N. Y. Acad. Sci..

[5] Temin H.M., Rubin H. (1958). Characteristics of an assay for Rous sarcoma virus and Rous sarcoma cells in tissue culture. Virology.

[6] Bather R. (1957). The nucleic acid of partially purified Rous No. I sarcoma virus. Br. J. Cancer.

[7] Rubin H., Temin H.M. (1959). A radiological study of cell-virus interaction in the Rous sarcoma. Virology.

[8] Temin H.M. (1964). The Participation of DNA in Rous Sarcoma Virus Production. Virology.

[9] Svoboda J., Chyle P., Simkovic D., Hilgert I. (1963). Demonstration of the absence of infectious Rous virus in rat tumour XC, whose structurally intact cells produce Rous sarcoma when transferred to chicks. Folia Biol..

[10] Baltimore D. (1970). RNA-dependent DNA polymerase in virions of RNA tumour viruses. Nature.

[11] Temin H.M., Mizutani S. (1970). RNA-dependent DNA polymerase in virions of Rous sarcoma virus. Nature.

[12] Stehelin D., Varmus H.E., Bishop J.M., Vogt P.K. (1976). DNA related to the transforming gene(s) of avian sarcoma viruses is present in normal avian DNA. Nature.

[13] Hayward W.S., Neel B.G., Astrin S.M. (1981). Activation of a cellular onc gene by promoter insertion in ALV-induced lymphoid leukosis. Nature.

[14] Neel B.G., Hayward W.S., Robinson H.L., Fang J., Astrin S.M. (1981). Avian leukosis virus-induced tumors have common proviral integration sites and synthesize discrete new RNAs: Oncogenesis by promoter insertion. Cell.

[15] Mann R., Mulligan R.C., Baltimore D. (1983). Construction of a retrovirus packaging mutant and its use to produce helper-free defective retrovirus. Cell.

[16] Williams D.A., Lemischka I.R., Nathan D.G., Mulligan R.C. (1984). Introduction of new genetic material into pluripotent haematopoietic stem cells of the mouse. Nature.

[17] Dick J.E., Magli M.C., Huszar D., Phillips R.A., Bernstein A. (1985). Introduction of a selectable gene into primitive stem cells capable of long-term reconstitution of the hemopoietic system of W/Wv mice. Cell.

[18] Kohn D.B. (2010). Update on gene therapy for immunodeficiencies. Clin. Immunol..

[19] Ott M.G., Schmidt M., Schwarzwaelder K., Stein S., Siler U., Koehl U., Glimm H., Kuhlcke K., Schilz A., Kunkel H. (2006). Correction of X-linked chronic granulomatous disease by gene therapy, augmented by insertional activation of MDS1-EVI1, PRDM16 or SETBP1. Nat. Med..

[20] Stein S., Ott M.G., Schultze-Strasser S., Jauch A., Burwinkel B., Kinner A., Schmidt M., Kramer A., Schwable J., Glimm H. (2010). Genomic instability and myelodysplasia with monosomy 7 consequent to EVI1 activation after gene therapy for chronic granulomatous disease. Nat. Med..

[21] Hacein-Bey-Abina S., Von Kalle C., Schmidt M., McCormack M.P., Wulffraat N., Leboulch P., Lim A., Osborne C.S., Pawliuk R., Morillon E. (2003). LMO2-associated clonal T cell proliferation in two patients after gene therapy for SCID-X1. Science.

[22] Hacein-Bey-Abina S., Garrigue A., Wang G.P., Soulier J., Lim A., Morillon E., Clappier E., Caccavelli L., Delabesse E., Beldjord K. (2008). Insertional oncogenesis in 4 patients after retrovirus-mediated gene therapy of SCID-X1. J. Clin. Invest..

[23] Howe S.J., Mansour M.R., Schwarzwaelder K., Bartholomae C., Hubank M., Kempski H., Brugman M.H., Pike-Overzet K., Chatters S.J., de Ridder D. (2008). Insertional mutagenesis combined with acquired somatic mutations causes leukemogenesis following gene therapy of SCID-X1 patients. J. Clin Invest..

[24] Braun C.J., Boztug K., Paruzynski A., Witzel M., Schwarzer A., Rothe M., Modlich U., Beier R., Gohring G., Steinemann D. (2014). Gene therapy for Wiskott-Aldrich syndrome—Long-term efficacy and genotoxicity. Sci. Transl. Med..

[25] Lazo P.A., Lee J.S., Tsichlis P.N. (1990). Long-distance activation of the Myc protooncogene by provirus insertion in Mlvi-1 or Mlvi-4 in rat T-cell lymphomas. Proc. Natl. Acad. Sci. USA.

[26] Bartholomew C., Ihle J.N. (1991). Retroviral insertions 90 kilobases proximal to the Evi-1 myeloid transforming gene activate transcription from the normal promoter. Mol. Cell Biol..

[27] Montini E., Cesana D., Schmidt M., Sanvito F., Bartholomae C.C., Ranzani M., Benedicenti F., Sergi L.S., Ambrosi A., Ponzoni M. (2009). The genotoxic potential of retroviral vectors is strongly modulated by vector design and integration site selection in a mouse model of HSC gene therapy. J. Clin. Invest..

[28] Heckl D., Schwarzer A., Haemmerle R., Steinemann D., Rudolph C., Skawran B., Knoess S., Krause J., Li Z., Schlegelberger B. (2012). Lentiviral vector induced insertional haploinsufficiency of Ebf1 causes murine leukemia. Mol. Ther..

[29] Cavazzana-Calvo M., Payen E., Negre O., Wang G., Hehir K., Fusil F., Down J., Denaro M., Brady T., Westerman K. (2010). Transfusion independence and HMGA2 activation after gene therapy of human beta-thalassaemia. Nature.

[30] Sokol M., Wabl M., Ruiz I.R., Pedersen F.S. (2014). Novel principles of gamma-retroviral insertional transcription activation in murine leukemia virus-induced end-stage tumors. Retrovirology.

[31] Mooslehner K., Karls U., Harbers K. (1990). Retroviral integration sites in transgenic Mov mice frequently map in the vicinity of transcribed DNA regions. J. Virol..

[32] Scherdin U., Rhodes K., Breindl M. (1990). Transcriptionally active genome regions are preferred targets for retrovirus integration. J. Virol..

[33] Schroder A.R., Shinn P., Chen H., Berry C., Ecker J.R., Bushman F. (2002). HIV-1 integration in the human genome favors active genes and local hotspots. Cell.

[34] Wu X., Li Y., Crise B., Burgess S.M. (2003). Transcription start regions in the human genome are favored targets for MLV integration. Science.

[35] Hematti P., Hong B.K., Ferguson C., Adler R., Hanawa H., Sellers S., Holt I.E., Eckfeldt C.E., Sharma Y., Schmidt M. (2004). Distinct genomic integration of MLV and SIV vectors in primate hematopoietic stem and progenitor cells. PLoS Biol..

[36] Barr S.D., Leipzig J., Shinn P., Ecker J.R., Bushman F.D. (2005). Integration targeting by avian sarcoma-leukosis virus and human immunodeficiency virus in the chicken genome. J. Virol..

[37] Wagner W., Laufs S., Blake J., Schwager C., Wu X., Zeller J.W., Ho A.D., Fruehauf S. (2005). Retroviral integration sites correlate with expressed genes in hematopoietic stem cells. Stem Cells.

[38] Beard B.C., Dickerson D., Beebe K., Gooch C., Fletcher J., Okbinoglu T., Miller D.G., Jacobs M.A., Kaul R., Kiem H.P. (2007). Comparison of HIV-derived lentiviral and MLV-based gammaretroviral vector integration sites in primate repopulating cells. Mol. Ther..

[39] Beard B.C., Keyser K.A., Trobridge G.D., Peterson L.J., Miller D.G., Jacobs M., Kaul R., Kiem H.P. (2007). Unique integration profiles in a canine model of long-term repopulating cells transduced with gammaretrovirus, lentivirus, or foamy virus. Hum. Gene Ther..

[40] Cattoglio C., Facchini G., Sartori D., Antonelli A., Miccio A., Cassani B., Schmidt M., von Kalle C., Howe S., Thrasher A.J. (2007). Hot spots of retroviral integration in human CD34+ hematopoietic cells. Blood.

[41] Brady T., Agosto L.M., Malani N., Berry C.C., O’Doherty U., Bushman F. (2009). HIV integration site distributions in resting and activated CD4+ T cells infected in culture. Aids.

[42] Shun M.C., Raghavendra N.K., Vandegraaff N., Daigle J.E., Hughes S., Kellam P., Cherepanov P., Engelman A. (2007). LEDGF/p75 functions downstream from preintegration complex formation to effect gene-specific HIV-1 integration. Genes Dev..

[43] Ciuffi A., Llano M., Poeschla E., Hoffmann C., Leipzig J., Shinn P., Ecker J.R., Bushman F. (2005). A role for LEDGF/p75 in targeting HIV DNA integration. Nat. Med..

[44] Marshall H.M., Ronen K., Berry C., Llano M., Sutherland H., Saenz D., Bickmore W., Poeschla E., Bushman F.D. (2007). Role of PSIP1/LEDGF/p75 in lentiviral infectivity and integration targeting. PLoS One.

[45] Cherepanov P., Maertens G., Proost P., Devreese B., Van Beeumen J., Engelborghs Y., de Clercq E., Debyser Z. (2003). HIV-1 integrase forms stable tetramers and associates with LEDGF/p75 protein in human cells. J. Biol. Chem.

[46] Llano M., Vanegas M., Fregoso O., Saenz D., Chung S., Peretz M., Poeschla E.M. (2004). LEDGF/p75 determines cellular trafficking of diverse lentiviral but not murine oncoretroviral integrase proteins and is a component of functional lentiviral preintegration complexes. J. Virol..

[47] Sharma A., Larue R.C., Plumb M.R., Malani N., Male F., Slaughter A., Kessl J.J., Shkriabai N., Coward E., Aiyer S.S. (2013). BET proteins promote efficient murine leukemia virus integration at transcription start sites. Proc. Natl. Acad. Sci. USA.

[48] Gupta S.S., Maetzig T., Maertens G.N., Sharif A., Rothe M., Weidner-Glunde M., Galla M., Schambach A., Cherepanov P., Schulz T.F. (2013). Bromo- and extraterminal domain chromatin regulators serve as cofactors for murine leukemia virus integration. J. Virol..

[49] De Rijck J., de Kogel C., Demeulemeester J., Vets S., El Ashkar S., Malani N., Bushman F.D., Landuyt B., Husson S.J., Busschots K. (2013). The bet family of proteins targets moloney murine leukemia virus integration near transcription start sites. Cell. Rep..

[50] Gijsbers R., Ronen K., Vets S., Malani N., de Rijck J., McNeely M., Bushman F.D., Debyser Z. (2010). LEDGF hybrids efficiently retarget lentiviral integration into heterochromatin. Mol. Ther..

[51] Vets S., De Rijck J., Brendel C., Grez M., Bushman F., Debyser Z., Gijsbers R. (2013). Transient Expression of an LEDGF/p75 Chimera Retargets Lentivector Integration and Functionally Rescues in a Model for X-CGD. Mol. Ther. Nucleic Acids.

[52] Hare S., Shun M.C., Gupta S.S., Valkov E., Engelman A., Cherepanov P. (2009). A novel co-crystal structure affords the design of gain-of-function lentiviral integrase mutants in the presence of modified PSIP1/LEDGF/p75. PLoS Pathog..

[53] Ciuffi A., Diamond T.L., Hwang Y., Marshall H.M., Bushman F.D. (2006). Modulating target site selection during human immunodeficiency virus DNA integration *in vitro* with an engineered tethering factor. Hum. Gene Ther..

[54] Ferris A.L., Wu X., Hughes C.M., Stewart C., Smith S.J., Milne T.A., Wang G.G., Shun M.C., Allis C.D., Engelman A. (2010). Lens epithelium-derived growth factor fusion proteins redirect HIV-1 DNA integration. Proc. Natl. Acad. Sci. USA.

[55] Aiyer S., Swapna G.V., Malani N., Aramini J.M., Schneider W.M., Plumb M.R., Ghanem M., Larue R.C., Sharma A., Studamire B. (2014). Altering murine leukemia virus integration through disruption of the integrase and BET protein family interaction. Nucleic Acids Res..

[56] El Ashkar S., de Rijck J., Demeulemeester J., Vets S., Madlala P., Cermakova K., Debyser Z., Gijsbers R. (2014). BET-independent MLV-based vectors target away from promoters and regulatory elements. Mol. Ther. Nucleic Acids.

[57] Mitchell R.S., Beitzel B.F., Schroder A.R., Shinn P., Chen H., Berry C.C., Ecker J.R., Bushman F.D. (2004). Retroviral DNA Integration: ASLV, HIV, and MLV Show Distinct Target Site Preferences. PLoS Biol..

[58] Narezkina A., Taganov K.D., Litwin S., Stoyanova R., Hayashi J., Seeger C., Skalka A.M., Katz R.A. (2004). Genome-wide analyses of avian sarcoma virus integration sites. J. Virol..

[59] Hu J., Renaud G., Gomes T.J., Ferris A., Hendrie P.C., Donahue R.E., Hughes S.H., Wolfsberg T.G., Russell D.W., Dunbar C.E. (2008). Reduced genotoxicity of avian sarcoma leukosis virus vectors in rhesus long-term repopulating cells compared to standard murine retrovirus vectors. Mol. Ther..

[60] Suerth J.D., Maetzig T., Brugman M.H., Heinz N., Appelt J.U., Kaufmann K.B., Schmidt M., Grez M., Modlich U., Baum C. (2012). Alpharetroviral self-inactivating vectors: Long-term transgene expression in murine hematopoietic cells and low genotoxicity. Mol. Ther..

[61] Moiani A., Suerth J.D., Gandolfi F., Rizzi E., Severgnini M., de Bellis G., Schambach A., Mavilio F. (2014). Genome-wide analysis of alpharetroviral integration in human hematopoietic stem/progenitor cells. Genes.

[62] Donahue R.E., Kessler S.W., Bodine D., McDonagh K., Dunbar C., Goodman S., Agricola B., Byrne E., Raffeld M., Moen R. (1992). Helper virus induced T cell lymphoma in nonhuman primates after retroviral mediated gene transfer. J. Exp. Med..

[63] Cesana D., Ranzani M., Volpin M., Bartholomae C., Duros C., Artus A., Merella S., Benedicenti F., Sergi Sergi L., Sanvito F. (2014). Uncovering and dissecting the genotoxicity of self-inactivating lentiviral vectors *in vivo*. Mol. Ther..

[64] Watanabe S., Temin H.M. (1983). Construction of a helper cell line for avian reticuloendotheliosis virus cloning vectors. Mol. Cell Biol..

[65] Miller A.D., Rosman G.J. (1989). Improved retroviral vectors for gene transfer and expression. Biotechniques.

[66] Soneoka Y., Cannon P.M., Ramsdale E.E., Griffiths J.C., Romano G., Kingsman S.M., Kingsman A.J. (1995). A transient three-plasmid expression system for the production of high titer retroviral vectors. Nucleic Acids Res..

[67] Yu S.F., von Ruden T., Kantoff P.W., Garber C., Seiberg M., Ruther U., Anderson W.F., Wagner E.F., Gilboa E. (1986). Self-inactivating retroviral vectors designed for transfer of whole genes into mammalian cells. Proc. Natl. Acad. Sci. USA.

[68] Zufferey R., Dull T., Mandel R.J., Bukovsky A., Quiroz D., Naldini L., Trono D. (1998). Self-inactivating lentivirus vector for safe and efficient *in vivo* gene delivery. J. Virol..

[69] Modlich U., Bohne J., Schmidt M., von Kalle C., Knoss S., Schambach A., Baum C. (2006). Cell-culture assays reveal the importance of retroviral vector design for insertional genotoxicity. Blood.

[70] Zychlinski D., Schambach A., Modlich U., Maetzig T., Meyer J., Grassman E., Mishra A., Baum C. (2008). Physiological promoters reduce the genotoxic risk of integrating gene vectors. Mol. Ther..

[71] Baum C., Dullmann J., Li Z., Fehse B., Meyer J., Williams D.A., von Kalle C. (2003). Side effects of retroviral gene transfer into hematopoietic stem cells. Blood.

[72] Lamers C.H., Willemsen R., van Elzakker P., van Steenbergen-Langeveld S., Broertjes M., Oosterwijk-Wakka J., Oosterwijk E., Sleijfer S., Debets R., Gratama J.W. (2011). Immune responses to transgene and retroviral vector in patients treated with *ex vivo*-engineered T cells. Blood.

[73] Kondo E., Akatsuka Y., Nawa A., Kuzushima K., Tsujimura K., Tanimoto M., Kodera Y., Morishima Y., Kuzuya K., Takahashi T. (2005). Retroviral vector backbone immunogenicity: Identification of cytotoxic t-cell epitopes in retroviral vector-packaging sequences. Gene Ther..

[74] Federspiel M.J., Hughes S.H. (1997). Retroviral gene delivery. Methods Cell Biol..

[75] Hughes S.H. (2004). The RCAS vector system. Folia Biol..

[76] Svoboda J. (1998). Molecular biology of cell nonpermissiveness to retroviruses. Has the time come?. Gene.

[77] Hu J., Ferris A., Larochelle A., Krouse A.E., Metzger M.E., Donahue R.E., Hughes S.H., Dunbar C.E. (2007). Transduction of rhesus macaque hematopoietic stem and progenitor cells with avian sarcoma and leukosis virus vectors. Hum. Gene Ther..

[78] Dong J., Roth M.G., Hunter E. (1992). A chimeric avian retrovirus containing the influenza virus hemagglutinin gene has an expanded host range. J. Virol..

[79] Barsov E.V., Hughes S.H. (1996). Gene transfer into mammalian cells by a Rous sarcoma virus-based retroviral vector with the host range of the amphotropic murine leukemia virus. J. Virol..

[80] Dull T., Zufferey R., Kelly M., Mandel R.J., Nguyen M., Trono D., Naldini L. (1998). A third-generation lentivirus vector with a conditional packaging system. J. Virol..

[81] Kraunus J., Schaumann D.H.S., Meyer J., Modlich U., Fehse B., Brandenburg G., von Laer D., Klump H., Schambach A., Bohne J. (2004). Self-inactivating retroviral vectors with improved RNA processing. Gene Therapy.

[82] Schambach A., Mueller D., Galla M., Verstegen M.M., Wagemaker G., Loew R., Baum C., Bohne J. (2006). Overcoming promoter competition in packaging cells improves production of self-inactivating retroviral vectors. Gene Ther..

[83] Suerth J.D., Maetzig T., Galla M., Baum C., Schambach A. (2010). Self-inactivating alpharetroviral vectors with a split-packaging design. J. Virol..

[84] Ruddell A. (1995). Transcription regulatory elements of the avian retroviral long terminal repeat. Virology.

[85] Modlich U., Navarro S., Zychlinski D., Maetzig T., Knoess S., Brugman M.H., Schambach A., Charrier S., Galy A., Thrasher A.J. (2009). Insertional transformation of hematopoietic cells by self-inactivating lentiviral and gammaretroviral vectors. Mol. Ther..

[86] Xu W., Russ J.L., Eiden M.V. (2012). Evaluation of residual promoter activity in gamma-retroviral self-inactivating (SIN) vectors. Mol. Ther..

[87] Kaufmann K.B., Brendel C., Suerth J.D., Mueller-Kuller U., Chen-Wichmann L., Schwable J., Pahujani S., Kunkel H., Schambach A., Baum C. (2012). Alpharetroviral vector-mediated gene therapy for X-CGD: Functional correction and lack of aberrant splicing. Mol. Ther..

[88] Yoder J.A., Walsh C.P., Bestor T.H. (1997). Cytosine methylation and the ecology of intragenomic parasites. Trends Genet..

[89] Barklis E., Mulligan R.C., Jaenisch R. (1986). Chromosomal position or virus mutation permits retrovirus expression in embryonal carcinoma cells. Cell.

[90] Flanagan J.R., Krieg A.M., Max E.E., Khan A.S. (1989). Negative control region at the 5' end of murine leukemia virus long terminal repeats. Mol. Cell Biol..

[91] Ellis J. (2005). Silencing and variegation of gammaretrovirus and lentivirus vectors. Hum. Gene Ther..

[92] Greger J.G., Katz R.A., Ishov A.M., Maul G.G., Skalka A.M. (2005). The cellular protein daxx interacts with avian sarcoma virus integrase and viral DNA to repress viral transcription. J. Virol..

[93] Poleshko A., Palagin I., Zhang R., Boimel P., Castagna C., Adams P.D., Skalka A.M., Katz R.A. (2008). Identification of cellular proteins that maintain retroviral epigenetic silencing: Evidence for an antiviral response. J. Virol..

[94] Katz R.A., Jack-Scott E., Narezkina A., Palagin I., Boimel P., Kulkosky J., Nicolas E., Greger J.G., Skalka A.M. (2007). High-frequency epigenetic repression and silencing of retroviruses can be antagonized by histone deacetylase inhibitors and transcriptional activators, but uniform reactivation in cell clones is restricted by additional mechanisms. J. Virol..

[95] Shalginskikh N., Poleshko A., Skalka A.M., Katz R.A. (2013). Retroviral DNA methylation and epigenetic repression are mediated by the antiviral host protein Daxx. J. Virol..

[96] Hejnar J., Plachy J., Geryk J., Machon O., Trejbalova K., Guntaka R.V., Svoboda J. (1999). Inhibition of the rous sarcoma virus long terminal repeat-driven transcription by *in vitro* methylation: Different sensitivity in permissive chicken cells *versus* mammalian cells. Virology.

[97] Hejnar J., Svoboda J., Geryk J., Fincham V.J., Hak R. (1994). High rate of morphological reversion in tumor cell line H-19 associated with permanent transcriptional suppression of the LTR, v-src, LTR provirus. Cell Growth Differ..

[98] Searle S., Gillespie D.A., Chiswell D.J., Wyke J.A. (1984). Analysis of the variations in proviral cytosine methylation that accompany transformation and morphological reversion in a line of Rous sarcoma virus-infected Rat-1 cells. Nucleic Acids Res..

[99] Jahner D., Stuhlmann H., Stewart C.L., Harbers K., Lohler J., Simon I., Jaenisch R. (1982). *De novo* methylation and expression of retroviral genomes during mouse embryogenesis. Nature.

[100] Klug C.A., Cheshier S., Weissman I.L. (2000). Inactivation of a GFP retrovirus occurs at multiple levels in long-term repopulating stem cells and their differentiated progeny. Blood.

[101] Hoeben R.C., Migchielsen A.A., van der Jagt R.C., van Ormondt H., van der Eb A.J. (1991). Inactivation of the Moloney murine leukemia virus long terminal repeat in murine fibroblast cell lines is associated with methylation and dependent on its chromosomal position. J. Virol..

[102] Plachy J., Kotab J., Divina P., Reinisova M., Senigl F., Hejnar J. (2010). Proviruses selected for high and stable expression of transduced genes accumulate in broadly transcribed genome areas. J. Virol..

[103] Senigl F., Auxt M., Hejnar J. (2012). Transcriptional provirus silencing as a crosstalk of *de novo* DNA methylation and epigenomic features at the integration site. Nucleic Acids Res..

[104] Antoniou M.N., Skipper K.A., Anakok O. (2013). Optimizing retroviral gene expression for effective therapies. Hum. Gene Ther..

[105] Benabdellah K., Gutierrez-Guerrero A., Cobo M., Munoz P., Martin F. (2014). A chimeric HS4-SAR insulator (IS2) that prevents silencing and enhances expression of lentiviral vectors in pluripotent stem cells. PLoS One.

[106] Senigl F., Plachy J., Hejnar J. (2008). The core element of a CpG island protects avian sarcoma and leukosis virus-derived vectors from transcriptional silencing. J. Virol..

[107] Hejnar J., Hajkova P., Plachy J., Elleder D., Stepanets V., Svoboda J. (2001). CpG island protects Rous sarcoma virus-derived vectors integrated into nonpermissive cells from DNA methylation and transcriptional suppression. Proc. Natl. Acad. Sci. USA.

[108] Merten O.W. (2004). State-of-the-art of the production of retroviral vectors. J. Gene Med..

[109] Coroadinha A.S., Gama-Norton L., Amaral A.I., Hauser H., Alves P.M., Cruz P.E. (2010). Production of retroviral vectors: Review. Curr. Gene Ther..

[110] Chong H., Starkey W., Vile R.G. (1998). A replication-competent retrovirus arising from a split-function packaging cell line was generated by recombination events between the vector, one of the packaging constructs, and endogenous retroviral sequences. J. Virol..

[111] U.S. Food and Drug Administration Guidance for Industry: Guidance for Human Somatic Cell Therapy and Gene Therapy. http://www.fda.gov/biologicsbloodvaccines/guidancecomplianceregulatoryinformation/guidances/cellularandgenetherapy/ucm072987.htm.

[112] Ni Y., Sun S., Oparaocha I., Humeau L., Davis B., Cohen R., Binder G., Chang Y.N., Slepushkin V., Dropulic B. (2005). Generation of a packaging cell line for prolonged large-scale production of high-titer HIV-1-based lentiviral vector. J. Gene Med..

[113] Otto E., Jones-Trower A., Vanin E.F., Stambaugh K., Mueller S.N., Anderson W.F., McGarrity G.J. (1994). Characterization of a replication-competent retrovirus resulting from recombination of packaging and vector sequences. Hum. Gene Ther..

[114] Von Werder A., Seidler B., Schmid R.M., Schneider G., Saur D. (2012). Production of avian retroviruses and tissue-specific somatic retroviral gene transfer *in vivo* using the RCAS/TVA system. Nat. Protoc..

[115] Bear A.S., Morgan R.A., Cornetta K., June C.H., Binder-Scholl G., Dudley M.E., Feldman S.A., Rosenberg S.A., Shurtleff S.A., Rooney C.M. (2012). Replication-competent retroviruses in gene-modified T cells used in clinical trials: Is it time to revise the testing requirements?. Mol. Ther..

[116] Ikeda Y., Takeuchi Y., Martin F., Cosset F.L., Mitrophanous K., Collins M. (2003). Continuous high-titer HIV-1 vector production. Nat. Biotechnol..

[117] Cosset F.L., Legras C., Chebloune Y., Savatier P., Thoraval P., Thomas J.L., Samarut J., Nigon V.M., Verdier G. (1990). A new avian leukosis virus-based packaging cell line that uses two separate transcomplementing helper genomes. J. Virol..

[118] Schaefer-Klein J., Givol I., Barsov E.V., Whitcomb J.M., VanBrocklin M., Foster D.N., Federspiel M.J., Hughes S.H. (1998). The EV-O-derived cell line DF-1 supports the efficient replication of avian leukosis-sarcoma viruses and vectors. Virology.

[119] Himly M., Foster D.N., Bottoli I., Iacovoni J.S., Vogt P.K. (1998). The DF-1 chicken fibroblast cell line: Transformation induced by diverse oncogenes and cell death resulting from infection by avian leukosis viruses. Virology.

[120] Loftus S.K., Larson D.M., Watkins-Chow D., Church D.M., Pavan W.J. (2001). Generation of RCAS vectors useful for functional genomic analyses. DNA Res..

[121] Suerth J.D., Schambach A., Baum C. (2012). Genetic modification of lymphocytes by retrovirus-based vectors. Curr. Opin. Immunol..

[122] Hotta A., Saito Y., Kyogoku K., Kawabe Y., Nishijima K., Kamihira M., Iijima S. (2006). Characterization of transient expression system for retroviral vector production. J. Biosci. Bioeng..

[123] Sandrin V., Boson B., Salmon P., Gay W., Negre D., Le Grand R., Trono D., Cosset F.L. (2002). Lentiviral vectors pseudotyped with a modified RD114 envelope glycoprotein show increased stability in sera and augmented transduction of primary lymphocytes and CD34+ cells derived from human and nonhuman primates. Blood.

[124] Ghani K., Cottin S., Kamen A., Caruso M. (2007). Generation of a high-titer packaging cell line for the production of retroviral vectors in suspension and serum-free media. Gene Ther..

[125] Le Doux J.M., Morgan J.R., Snow R.G., Yarmush M.L. (1996). Proteoglycans secreted by packaging cell lines inhibit retrovirus infection. J. Virol..

[126] Suomalainen M., Garoff H. (1994). Incorporation of homologous and heterologous proteins into the envelope of Moloney murine leukemia virus. J. Virol..

[127] Cosset F.L., Takeuchi Y., Battini J.L., Weiss R.A., Collins M.K. (1995). High-titer packaging cells producing recombinant retroviruses resistant to human serum. J. Virol..

[128] Wicke D.C., Meyer J., Buesche G., Heckl D., Kreipe H., Li Z., Welte K.H., Ballmaier M., Baum C., Modlich U. (2010). Gene therapy of MPL deficiency: Challenging balance between leukemia and pancytopenia. Mol. Ther..

[129] Voelkel C., Galla M., Dannhauser P.N., Maetzig T., Sodeik B., Schambach A., Baum C. (2012). Pseudotype-independent nonspecific uptake of gammaretroviral and lentiviral particles in human cells. Hum. Gene Ther..

[130] Hacein-Bey-Abina S., Hauer J., Lim A., Picard C., Wang G.P., Berry C.C., Martinache C., Rieux-Laucat F., Latour S., Belohradsky B.H. (2010). Efficacy of gene therapy for X-linked severe combined immunodeficiency. N. Engl. J. Med..

[131] Gaspar H.B., Cooray S., Gilmour K.C., Parsley K.L., Adams S., Howe S.J., Al Ghonaium A., Bayford J., Brown L., Davies E.G. (2011). Long-term persistence of a polyclonal T cell repertoire after gene therapy for X-linked severe combined immunodeficiency. Sci. Transl. Med..

[132] Aiuti A., Cattaneo F., Galimberti S., Benninghoff U., Cassani B., Callegaro L., Scaramuzza S., Andolfi G., Mirolo M., Brigida I. (2009). Gene therapy for immunodeficiency due to adenosine deaminase deficiency. N. Engl. J. Med..

[133] Aiuti A., Biasco L., Scaramuzza S., Ferrua F., Cicalese M.P., Baricordi C., Dionisio F., Calabria A., Giannelli S., Castiello M.C. (2013). Lentiviral hematopoietic stem cell gene therapy in patients with Wiskott-Aldrich syndrome. Science.

[134] Cartier N., Hacein-Bey-Abina S., Bartholomae C.C., Veres G., Schmidt M., Kutschera I., Vidaud M., Abel U., Dal-Cortivo L., Caccavelli L. (2009). Hematopoietic stem cell gene therapy with a lentiviral vector in X-linked adrenoleukodystrophy. Science.

[135] Biffi A., Montini E., Lorioli L., Cesani M., Fumagalli F., Plati T., Baldoli C., Martino S., Calabria A., Canale S. (2013). Lentiviral hematopoietic stem cell gene therapy benefits metachromatic leukodystrophy. Science.

[136] Rosenberg S.A., Aebersold P., Cornetta K., Kasid A., Morgan R.A., Moen R., Karson E.M., Lotze M.T., Yang J.C., Topalian S.L. (1990). Gene transfer into humans—Immunotherapy of patients with advanced melanoma, using tumor-infiltrating lymphocytes modified by retroviral gene transduction. N Engl J. Med..

[137] Morgan R.A., Dudley M.E., Wunderlich J.R., Hughes M.S., Yang J.C., Sherry R.M., Royal R.E., Topalian S.L., Kammula U.S., Restifo N.P. (2006). Cancer regression in patients after transfer of genetically engineered lymphocytes. Science.

[138] Parkhurst M.R., Yang J.C., Langan R.C., Dudley M.E., Nathan D.A., Feldman S.A., Davis J.L., Morgan R.A., Merino M.J., Sherry R.M. (2011). T cells targeting carcinoembryonic antigen can mediate regression of metastatic colorectal cancer but induce severe transient colitis. Mol. Ther..

[139] Robbins P.F., Morgan R.A., Feldman S.A., Yang J.C., Sherry R.M., Dudley M.E., Wunderlich J.R., Nahvi A.V., Helman L.J., Mackall C.L. (2011). Tumor regression in patients with metastatic synovial cell sarcoma and melanoma using genetically engineered lymphocytes reactive with NY-ESO-1. J. Clin. Oncol..

[140] Hart D.P., Xue S.A., Thomas S., Cesco-Gaspere M., Tranter A., Willcox B., Lee S.P., Steven N., Morris E.C., Stauss H.J. (2008). Retroviral transfer of a dominant TCR prevents surface expression of a large proportion of the endogenous TCR repertoire in human T cells. Gene Ther..

[141] Dotti G., Gottschalk S., Savoldo B., Brenner M.K. (2014). Design and development of therapies using chimeric antigen receptor-expressing T cells. Immunol. Rev..

[142] Sadelain M., Brentjens R., Riviere I. (2013). The basic principles of chimeric antigen receptor design. Cancer Discov..

[143] Kalos M., Levine B.L., Porter D.L., Katz S., Grupp S.A., Bagg A., June C.H. (2011). T cells with chimeric antigen receptors have potent antitumor effects and can establish memory in patients with advanced leukemia. Sci. Transl. Med..

[144] Kochenderfer J.N., Wilson W.H., Janik J.E., Dudley M.E., Stetler-Stevenson M., Feldman S.A., Maric I., Raffeld M., Nathan D.A., Lanier B.J. (2010). Eradication of B-lineage cells and regression of lymphoma in a patient treated with autologous T cells genetically engineered to recognize CD19. Blood.

[145] Brentjens R.J., Riviere I., Park J.H., Davila M.L., Wang X., Stefanski J., Taylor C., Yeh R., Bartido S., Borquez-Ojeda O. (2011). Safety and persistence of adoptively transferred autologous CD19-targeted T cells in patients with relapsed or chemotherapy refractory B-cell leukemias. Blood.

[146] Louis C.U., Savoldo B., Dotti G., Pule M., Yvon E., Myers G.D., Rossig C., Russell H.V., Diouf O., Liu E. (2011). Antitumor activity and long-term fate of chimeric antigen receptor-positive T cells in patients with neuroblastoma. Blood.

[147] Bonini C., Ferrari G., Verzeletti S., Servida P., Zappone E., Ruggieri L., Ponzoni M., Rossini S., Mavilio F., Traversari C. (1997). HSV-TK gene transfer into donor lymphocytes for control of allogeneic graft-versus-leukemia. Science.

[148] Ciceri F., Bonini C., Stanghellini M.T., Bondanza A., Traversari C., Salomoni M., Turchetto L., Colombi S., Bernardi M., Peccatori J. (2009). Infusion of suicide-gene-engineered donor lymphocytes after family haploidentical haemopoietic stem-cell transplantation for leukaemia (the TK007 trial): a non-randomised phase I-II study. Lancet Oncol..

[149] Di Stasi A., Tey S.K., Dotti G., Fujita Y., Kennedy-Nasser A., Martinez C., Straathof K., Liu E., Durett A.G., Grilley B. (2011). Inducible apoptosis as a safety switch for adoptive cell therapy. N. Engl. J. Med..

[150] Scholler J., Brady T.L., Binder-Scholl G., Hwang W.T., Plesa G., Hege K.M., Vogel A.N., Kalos M., Riley J.L., Deeks S.G. (2012). Decade-long safety and function of retroviral-modified chimeric antigen receptor T cells. Sci. Transl. Med..

[151] Newrzela S., Al-Ghaili N., Heinrich T., Petkova M., Hartmann S., Rengstl B., Kumar A., Jack H.M., Gerdes S., Roeder I. (2012). T-cell receptor diversity prevents T-cell lymphoma development. Leukemia.

[152] Newrzela S., Cornils K., Li Z., Baum C., Brugman M.H., Hartmann M., Meyer J., Hartmann S., Hansmann M.L., Fehse B. (2008). Resistance of mature T cells to oncogene transformation. Blood.

[153] Heinrich T., Rengstl B., Muik A., Petkova M., Schmid F., Wistinghausen R., Warner K., Crispatzu G., Hansmann M.L., Herling M. (2013). Mature T-cell lymphomagenesis induced by retroviral insertional activation of janus kinase 1. Mol. Ther..

